# Organization of Presynaptic Autophagy-Related Processes

**DOI:** 10.3389/fnsyn.2022.829354

**Published:** 2022-03-17

**Authors:** Eckart D. Gundelfinger, Anna Karpova, Rainer Pielot, Craig C. Garner, Michael R. Kreutz

**Affiliations:** ^1^Research Group Neuroplasticity, Leibniz Institute for Neurobiology, Magdeburg, Germany; ^2^Institute of Pharmacology and Toxicology, Medical Faculty, Otto von Guericke University, Magdeburg, Germany; ^3^Center for Behavioral Brain Sciences (CBBS), Magdeburg, Germany; ^4^German Center for Neurodegenerative Diseases (DZNE), Berlin, Germany; ^5^Charité – Universitätsmedizin Berlin, Berlin, Germany; ^6^Center for Molecular Neurobiology (ZMNH), University Hospital Hamburg-Eppendorf, Hamburg, Germany; ^7^German Center for Neurodegenerative Diseases (DZNE), Magdeburg, Germany

**Keywords:** autophagy, endolysosomal system, active zone (AZ), Bassoon, endocytic zone, synaptic vesicle (SV), amphisome, presynaptic proteostasis

## Abstract

Brain synapses pose special challenges on the quality control of their protein machineries as they are far away from the neuronal soma, display a high potential for plastic adaptation and have a high energy demand to fulfill their physiological tasks. This applies in particular to the presynaptic part where neurotransmitter is released from synaptic vesicles, which in turn have to be recycled and refilled in a complex membrane trafficking cycle. Pathways to remove outdated and damaged proteins include the ubiquitin-proteasome system acting in the cytoplasm as well as membrane-associated endolysosomal and the autophagy systems. Here we focus on the latter systems and review what is known about the spatial organization of autophagy and endolysomal processes within the presynapse. We provide an inventory of which components of these degradative systems were found to be present in presynaptic boutons and where they might be anchored to the presynaptic apparatus. We identify three presynaptic structures reported to interact with known constituents of membrane-based protein-degradation pathways and therefore may serve as docking stations. These are (i) scaffolding proteins of the cytomatrix at the active zone, such as Bassoon or Clarinet, (ii) the endocytic machinery localized mainly at the peri-active zone, and (iii) synaptic vesicles. Finally, we sketch scenarios, how presynaptic autophagic cargos are tagged and recruited and which cellular mechanisms may govern membrane-associated protein turnover in the presynapse.

## Introduction

Brain synapses can have long lifetimes (e.g., Holtmaat et al., 2005; Qiao et al., 2016) and display an enormous potential for plasticity (e.g., Citri and Malenka, 2008; Yang and Calakos, 2013). They also have a very high energy demand to maintain their functions (e.g., Harris et al., 2012), a situation that poses additional metabolic stress on synaptic protein components and requires an efficient management of proteostasis. This applies in particular to the presynaptic compartment with its apparatus for regulated neurotransmitter release, which rapidly and efficiently recycles releasable neurotransmitter-filled synaptic vesicles (SVs). Biosynthesis of presynaptic components occurs predominantly in the neuronal soma, where they are packaged into specific precursor organelles and are actively transported along the axon to presynaptic sites (for a review see Rizalar et al., 2021). The lifespan of presynaptic proteins varies with half-lives ranging from a few hours to several days (Hakim et al., 2016; Fornasiero et al., 2018; Cohen and Ziv, 2019), which is very short compared to the lifespan of neurons and synapses. Hence, presynaptic proteins must be continuously replaced in a specific and highly coordinated manner.

**FIGURE 1:**
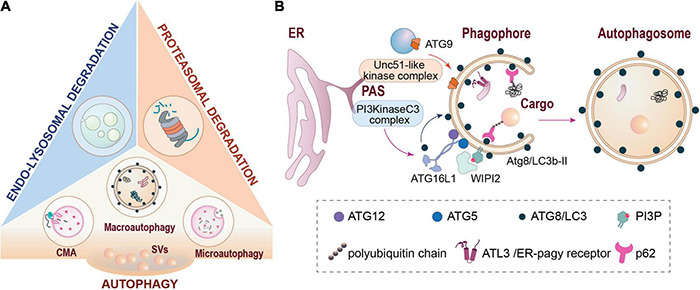
Major pathways of cellular proteostasis. **(A)** Major degradative pathways at the presynapse include proteasomal degradation, endo-lysosomal degradation and different types of autophagy. **(B)** Schematic representation of autophagosome formation and cargo recruitment into autophagosomes via autophagy receptors such as p62/SQMTS1 or ATL3. CMA, chaperone-mediated autophagy; ER, endoplasmic reticulum; PAS, phagophore assembly site (also designated as pre-autophagosomal structure); SVs, synaptic vesicles.

Three main systems are in place to mediate this turnover, i.e., the ubiquitin-proteasome system (UPS) acting in the cytoplasm (Rinetti and Schweizer, 2010; Lazarevic et al., 2011; Waites et al., 2013; Cohen and Ziv, 2017; Soykan et al., 2021), and the endolysosomal pathway and autophagy-related processes acting via degradative membranous organelles (Azarnia Tehran et al., 2018; Jin et al., 2018; Boecker and Holzbaur, 2019; Kuijpers et al., 2020; Lieberman and Sulzer, 2020; Andres-Alonso et al., 2021; Soykan et al., 2021; Figure 1A). Various autophagic pathways exist in parallel. These comprise macroautophagy, which can act in bulk or selective modes (including ER-phagy, aggrephagy and mitophagy) as well as chaperone-mediated autophagy (CMA) and microautophagy (Stavoe and Holzbaur, 2019). In this review, we will focus mainly on presynaptic macroautophagy, but will also consider constituents of other pathways of membrane-associated protein turnover. Macroautophagy (from here on referred to as autophagy) starts with the formation of a phagophore at a phagophore assembly site (PAS) and the recruitment of membranes from various sources via ATG9-containing vesicles (Figure 1B; Dikic and Elazar, 2018). Recruitment of cargo into the autophagosome is mediated via specific receptors/adaptors that bind ATG8-like proteins, which are anchored to the phagophore membrane via conjugation to phosphatidylethanolamine (e.g., Deng et al., 2017; Dikic and Elazar, 2018; Gatica et al., 2018). One major way of determining cargoes for autophagy is the conjugation of poly-ubiquitin chains, but there are also selective modes of autophagy that function independently of ubiquitination (Khaminets et al., 2016). On the other hand, ubiquitination is also involved in the tagging of proteins for proteasomal and endolysosomal degradation. This is achieved via several hundreds of E3-ubiquitin ligases encoded by mammalian genomes and makes them important surveyors of the various pathways of proteostasis (Wang and Le, 2019). In this regard, we will also address the question of which E3 ligases may mediate aspects of presynaptic autophagy.

## Autophagosome Biogenesis in Axons

In their axonal compartment, neurons entertain a steady process of basal autophagy (Figure 1B). Phagophore formation during basal autophagy is largely restricted to distal axons where autophagosomes are constitutively generated and then retrogradely transported toward the cell soma in a Dynein-dependent manner to fuse with lysosomes (Cheng et al., 2015; Maday and Holzbaur, 2016). Autophagy occurs at a basal level in all cells, but it can be up-regulated during stress, starvation, or infection. In neurons, however, conflicting reports have been published on whether neurons are sensitive (Boland et al., 2008; Nixon et al., 2008; Young et al., 2009; Alirezaei et al., 2010; Rubinsztein and Nixon, 2010; Hernandez et al., 2012; Catanese et al., 2018) or insensitive (Mizushima et al., 2004; Tsvetkov et al., 2010; Maday and Holzbaur, 2016; Kulkarni et al., 2020) to nutrient deprivation and/or mTOR inhibition. Likewise, the ordered recruitment of assembly factors for phagophore formation and maturation has been studied in much less detail for neuronal autophagy, but seems to be reminiscent of what has been found in non-neuronal cells (Maday and Holzbaur, 2014). The continuous formation of autophagosomes under steady-state conditions of neuronal signaling has led to the notion that autophagy is involved in homeostatic processes of proteostasis, particularly in axons. In fact, the primary membrane donor for the biogenesis of autophagosomes in distal axons is the endoplasmic reticulum (ER; Maday and Holzbaur, 2014). In very recent work, it was shown that the ER is also the main substrate for neuronal autophagy and that ER-phagy is important maintaining the integrity of axonal ER calcium stores and calcium release through ryanodine receptors (Kuijpers et al., 2021). In this contribution to the review series on ‘Molecular Nanomachines of the Presynaptic Terminal’, we will first examine which constituents of autophagic processes, as well as other interacting membrane-associated pathways of protein turnover, have been detected in presynaptic compartments to date, and how are they organized to fulfill their functions.

## Autophagy-Related and Endolysosomal Proteins in the Presynapse

**FIGURE 2:**
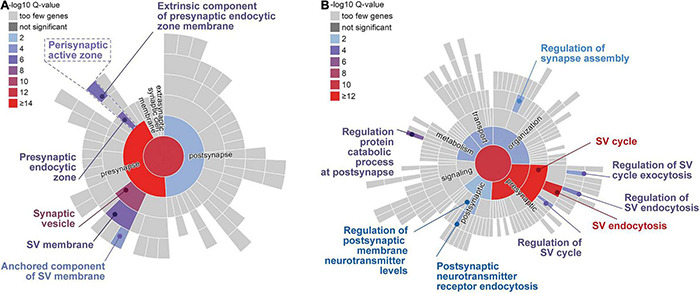
Sunburst plots of gene enrichment analyses for autophagy-related genes/proteins included in SynGO (Koopmans et al., 2019). Significantly enriched cellular components **(A)** and biological processes **(B)** are indicated by color code. The top-level terms of the Gene Ontology (GO) term tree are represented by the inner circle; the second level of the term tree is denoted by the innermost ring and so on. Presynaptic structures **(A)** and processes, like synaptic vesicle (SV) cycle and SV endocytosis **(B)** are significantly over-represented. Note, autophagy-related terms were not annotated in the database. Database entries for cellular components and for biological processes were considered as indicated in Table 1.

To assess the presence of components of the autophagic and the endolysosomal protein degradation systems as well as major elements contributing to chaperone-mediated autophagy (CMA) in brain synapses, we assembled, based on the relevant literature, a list of proteins contributing to membrane-based degradation pathways. More than 90 proteins and protein complexes were identified (Table 1), which were inspected for their localization in synapses and, in particular, in presynaptic compartments by examining whether they are included in relevant databases, i.e., the SynProt database of synaptic proteins^1^ (Pielot et al., 2012), and the synaptic gene ontology database SynGO, an expert-curated knowledge base of synaptic proteins^2^ (Koopmans et al., 2019). In addition, we checked whether their presence in the presynapse has been reported otherwise. One important source was a study on the so-called hidden proteome of SVs that identified dozens of SV-resident and SV-visitor proteins of the autophagic and endolysosomal pathways (Taoufiq et al., 2020). About 50% of the proteins listed in Table 1 are present in the SynProt databases, which include proteins identified in synaptic proteome studies (SynProt classics) and in studies on the proteomes of presynaptic compartments (PreProt). About one third of the proteins/protein complexes are found in SynGO, which provides a much higher and expert-evaluated resolution with respect to the compartmental localization of the identified proteins. In essence, we could assign many of the entries included in Table 1 to the presynaptic compartment. A closer inspection of these proteins allowed us to draw conclusions on the major presynaptic structures involved in the anchoring for the autophagic machinery. These include the cytomatrix at the active zone (CAZ) of neurotransmitter release, represented by the CAZ scaffolding protein Bassoon, the endocytic machinery mainly localized peripherally to the active zone (AZ), the peri-AZ, and SVs (Figure 2A). Many of the identified proteins that are included in SynGO can be functionally assigned to presynaptic biological processes related to the SV cycle and SV endocytosis (Figure 2B).

**TABLE 1:** Proteins related to autophagic and endolysosomal processes.

Protein	UniProt acc.# (name); alternative names	Present in SynProt^a^	Present in Hidden SV Proteome^b^	Present in SynGO^c^	Localization in Synapse^d^	Function/Remarks	Selected references
AMBRA1	A2AH22 (AMRA1_MOUSE); Activating molecule in BECN1-regulated autophagy protein 1	No	No	No	n.d.	Part of the autophagy nucleation complex	(Fimia et al., 2007; Dikic and Elazar, 2018; Tomoda et al., 2020)
Annexin A7	Q07076 (ANXA7_MOUSE), Annexin-7; Synexin	Yes	Yes SV-visitor	No	Synapse SV	Autophagy, promotes membrane fusion	(Taoufiq et al., 2020)
AP-2	P17426 (AP2A1_MOUSE), P17427 (AP2A2_MOUSE), Q9DBG3 (AP2B1_MOUSE), P84091 (AP2M1_MOUSE), P62743 (AP2S1_MOUSE); Adaptor protein AP-2 complex subunits mu, alpha-1, alpha-2, beta1 and sigma	Yes	Yes SV-resident	Yes	Synapse, Presynapse, Peri-AZ SV	Adaptor for Clathrin-mediated membrane fission. Together with CALM, AP-2 mediates formation of autophagosomes/signaling amphisomes; present in SV preparations.	(Boyken et al., 2013; Tian et al., 2014; Weingarten et al., 2014; Kononenko et al., 2017; Wang et al., 2017; Azarnia Tehran et al., 2018; Taoufiq et al., 2020)
Arf6	P62331 (ARF6_MOUSE); ADP-ribosylation factor 6;	Yes	Yes SV-visitor	Yes	Presynapse Postsynapse SV	Small GTPase antagonizes Rab35 in SV recycling; regulates autophagy by interplay with Synj1 and/or phospholipase D	(Moreau et al., 2012; George et al., 2016; Sheehan and Waites, 2019)
Arl8	Q9CQW2 (ARL8B_MOUSE); ADP-ribosylation factor-like protein 8B;	Yes (8A,B)	Yes (8A,B) SV-visitor	(Yes) BP only	Synapse, Presynapse SV, AZ	Present in synaptic vesicle (SV) and active zone (AZ) preparations; anterograde transport of lysosome-related vesicles	(Takamori et al., 2006; Boyken et al., 2013; Vukoja et al., 2018; Farfel-Becker et al., 2019; De Pace et al., 2020; Borchers et al., 2021)
ATG2a	Q6P4T0 (ATG2A_MOUSE); Autophagy-related protein 2 homolog A; ortholog: D3ZT64 (D3ZT64_RAT, formerly XP_219529)	Yes	(Yes) low abundance P2’-fraction	No	Synapse, Synaptosome Presynapse, SV	Present in SV protein preparation; Transfers phospholipids to the phagophore.	(Takamori et al., 2006) (Soukup et al., 2016; Sawa-Makarska et al., 2020; Chang et al., 2021)
ATG3	Q9CPX6 (ATG3_MOUSE); Autophagy-related protein 3. Ubiquitin-like-conjugating enzyme ATG3; Short name: APG3-like	No	P2’-fraction	No	Synapse	E2-like enzyme of the ubiquitin-like conjugation system; ATG3 can be recruited to membranes by EndoA	(Soukup et al., 2016; Vijayan and Verstreken, 2017; Dikic and Elazar, 2018; Hill and Colon-Ramos, 2020)
ATG4 family	Q9U1N6 (ATG42_CAEEL); Q8C9S8 (ATG4A_MOUSE) etc.; Cysteine proteases ATG4A-D	No	No	No	n.d.	Cysteine proteases of the ubiquitin-like conjugation system; *C. elegans*: A TG-4.2 involved in autophagosome clearance	(Dikic and Elazar, 2018; Hill et al., 2019; Hill and Colon-Ramos, 2020)
ATG5	Q99J83 (ATG5_MOUSE), Autophagy protein 5; APG5-like	No	No	No	Synapse, Presynapse	Part of the ATG12-ATG5-ATG16L1 E3-like complex of the Ubiquitin-like conjugation system; binds AZ protein Bassoon; colocalizes with presynaptic markers in primary neurons; ATG5-KO in neurons induces axonal ER-phagy.	(Vijayan and Verstreken, 2017; Dikic and Elazar, 2018; Tomoda et al., 2020; Andres-Alonso et al., 2021; Chang et al., 2021) (Okerlund et al., 2017; Kuijpers et al., 2021; Soykan et al., 2021)
ATG7	Q9D906 (ATG7_MOUSE); Ubiquitin-like modifier-activating enzyme ATG7; Autophagy-related protein 7; APG7-like	No	P2’-fraction	No	Synapse Synaptosome	E1-like enzyme of the Ubiquitin-like conjugation system; ATG7 deficiency has severe effects on presynaptic function.	(Komatsu et al., 2007; Vijayan and Verstreken, 2017; Dikic and Elazar, 2018; Lieberman and Sulzer, 2020; Overhoff et al., 2021)
ATG9A	Q68FE2 (ATG9A_MOUSE); Autophagy-related protein 9A; APG9-like 1;	Yes	Yes SV-resident	Yes	Synapse, Presynapse; SV, AZ, Peri-AZ	Lipid scramblase involved in autophagosome biogenesis; present in SV preparations (SV-resident repertoire); likely to be involved in SV autophagy.	(Boyken et al., 2013; Stavoe et al., 2016; Guardia et al., 2020; Hill and Colon-Ramos, 2020; Maeda et al., 2020; Sawa-Makarska et al., 2020; Chang et al., 2021)
ATG10	Q8R1P4 (ATG10_MOUSE); Ubiquitin-like-conjugating enzyme ATG10; Autophagy-related protein 10	No	No	No	n.d.	E2-like enzyme of the ubiquitin-like conjugation system; conjugates ATG12 on ATG5.	(Dikic and Elazar, 2018; Stavoe and Holzbaur, 2019)
ATG12	Q9CQY1 (ATG12_MOUSE); Ubiquitin-like protein ATG12; Autophagy-related protein 12; APG12-like	No	No	No	n.d.	Part of the ATG12-ATG5-ATG16L1 E3-like complex of the Ubiquitin-like conjugation system;	(Vijayan and Verstreken, 2017; Dikic and Elazar, 2018; Chang et al., 2021)
ATG13	Q91YI1 (ATG13_MOUSE); Autophagy-related protein 13	No	No	No	n.d.	Adaptor protein within the ULK/ATG1 complex	(Dikic and Elazar, 2018; Hill and Colon-Ramos, 2020; Tomoda et al., 2020)
ATG14/ATG14L	Q8CDJ3 (BAKOR_MOUSE) Beclin 1-associated autophagy-related key regulator; Autophagy-related protein 14-like protein	No	No	No	n.d.	Part of the PIK3C3 complex; and promotes autophagosome-endolysosome fusion.	(Diao et al., 2015; Dikic and Elazar, 2018; Hill and Colon-Ramos, 2019)
ATG16L1	Q8C0J2 (A16L1_MOUSE); Autophagy-related protein 16-1; APG16-like 1	Yes	Yes SV-visitor	No	Synapse; Presynapse, SV	Part of the ATG12-ATG5-ATG16L1 E3-like complex of Ub-like conjugation system; may be linked to SV autophagy.	(Binotti et al., 2015; Dikic and Elazar, 2018; Hill and Colon-Ramos, 2020; Chang et al., 2021)
ATG101	Q9D8Z6 (ATGA1_MOUSE); Q9VWQ1 (Q9VWQ1_DROME); Autophagy-related protein 101	No	P2’-fraction	No	Synapse Synaptosome	Part of the ULK/ATG1 complex	(Dikic and Elazar, 2018; Chang et al., 2021)
ATL2/3	Q6PA06 (ATLA2_MOUSE); Atlastin-2; ADP-ribosylation factor-like protein 6-interacting protein 2; Q91YH5 (ATLA3_MOUSE); Atlastin-3	Yes (ATL2)	Yes ATL2: SV-visitor ATL3: SV-visitor	No	Synapse, Presynapse SV	Recruitment and stabilization of ATG1 complex at the FIP200-ATG13–specified autophagosome formation sites on ER. Adaptor for ER-phagy; required for presynaptic function at larval NMJ.	(De Gregorio et al., 2017; Andres-Alonso et al., 2021; Liu et al., 2021; Wojnacki et al., 2021)
BAG3	Q9JLV1 (BAG3_MOUSE); BAG family molecular chaperone regulator 3; Bcl-2-associated athanogene 3	Yes	P2’-fraction	No	Synapse Synaptosome	Co-chaperonin for HSC70, interacts with synaptopodin	(Sturner and Behl, 2017; Ji et al., 2019)
Bassoon	O88737 (BSN_MOUSE); AZ scaffold protein Bassoon.	Yes	Yes SV-visitor	Yes	Presynapse, AZ, SV	Recruits ATG5 to the presynaptic AZ; functionally interacts with Parkin.	(Okerlund et al., 2017; Vijayan and Verstreken, 2017; Hill and Colon-Ramos, 2020; Hoffmann-Conaway et al., 2020; Andres-Alonso et al., 2021)
Beclin-1/ATG6	O88597 (BECN1_MOUSE), Beclin-1	No	Yes SV-visitor	No	Presynape SV	Core subunit of the PI3KinaseC3 complex; regulates Vps34 lipid kinase;	(Dikic and Elazar, 2018)
CALM/PICALM	Q7M6Y3 (PICAL_MOUSE); Phosphatidylinositol-binding Clathrin assembly protein; Clathrin assembly lymphoid myeloid leukemia	Yes	Yes SV-visitor	Yes	Presynapse; Peri-AZ, SV	Autophagic sorting adaptor; endocytic adaptor;	(Takamori et al., 2006; Tian et al., 2013; Azarnia Tehran et al., 2018)
CISD2	Q9CQB5 (CISD2_MOUSE); CDGSH iron-sulfur domain-containing protein 2; Miner 1; NAF-1	No	Yes SV-visitor	No	Presynapse SV	Regulator of autophagy; contributes to control of Beclin-1.	(Chang et al., 2010; Shen et al., 2021)
DFCP1/Zfyve1	Q810J8 (ZFYV1_MOUSE); Zinc finger FYVE domain-containing protein 1	No	Yes SV-visitor	No	Presynapse SV	PI3P- binding protein; enriched in omegasomes of the ER	(Dikic and Elazar, 2018; Hill and Colon-Ramos, 2020)
DENND3	A2RT67 (DEND3_MOUSE); DENN domain-containing protein 3	No	No	No	n.d.	Actin-binding guanine nucleotide exchange factor for Rab12 activated by ULK1 and required for autophagy	(Xu et al., 2015, 2018; Wojnacki and Galli, 2018; Wei and Duan, 2019)
Endophilin-A/EndoA	Q62420 (SH3G2_MOUSE); Endophilin A1, A2; Q8T390 (SH3G3_DROME); Endophilin-A; SH3 domain-containing GRB2-like protein;	Yes	Yes SV-visitor	Yes	Presynapse; SV Peri-AZ	Endocytic adaptor essential for SV recycling; forms docking stations for autophagic proteins at synapses.	(Murdoch et al., 2016; Soukup et al., 2016, 2018; Vijayan and Verstreken, 2017; Azarnia Tehran et al., 2018)
Endophilin-B1	Q9JK48 (SHLB1_MOUSE); SH3 domain-containing GRB2-like protein B1; BIF-1	No	Yes SV-visitor	No	Presynapse, SV,	Associates with PI3KC3-C2 and regulates ATG9a trafficking	(Takahashi et al., 2011; Taoufiq et al., 2020)
FADD	Q61160 (FADD_MOUSE); FAS-associated death domain protein	No	No	No	n.d.	Death domain protein that directly interacts with ATG5,	(Pyo et al., 2005)
FAM134B	Q8VE91 (RETR1_MOUSE); Reticulophagy regulator 1; family with sequence similarity 134 member B;	No	No	No	n.d.	Adaptor for ER-phagy	(Khaminets et al., 2015; Stavoe and Holzbaur, 2019; Andres-Alonso et al., 2021)
FIP200/Rb1cc1	Q9ESK9 (RBCC1_MOUSE); RB1-inducible coiled-coil protein 1; FAK family kinase-interacting protein of 200 kDa	Yes	P2’-fraction	No	Synapse, Synaptosome	Part of the ULK/ATG1 complex	(Dikic and Elazar, 2018; Lieberman and Sulzer, 2020; Tomoda et al., 2020)
FBXO32	Q9CPU7 (FBX32_MOUSE); F-box only protein 32; Atrogin-1	No	No	No	n.d.	E3-ubiquitin ligase; interacts with endophilin-A to control autophagosome formation and protein homeostasis.	(Murdoch et al., 2016; Azarnia Tehran et al., 2018)
GABARAPs/ ATG8-like	Q9DCD6 (GBRAP_MOUSE); GABA receptor associated protein; Q8R3R8 (GBRL1_MOUSE); P60521 (GBRL2_MOUSE); GABARP-like 1, 2; LGG-1 and LGG-2 in *C. elegans*	Yes GABARAPL1 GABARAPL2	GABARAP, GABARAPL1, GABARAPL2 P2’-fraction	No	Synapse, Synaptosome Presynapse	Lipidated ATG8-like proteins that are key factors for various autophagic processes. In *C. elegans* localized in presynapse	(Dikic and Elazar, 2018; Hill et al., 2019; Martens and Fracchiolla, 2020)
HSC70	P63017 (HSP7C_MOUSE); Heat shock cognate 71 kDa protein; Heat shock 70 kDa protein 8 (Hspa8)	Yes	Yes SV-visitor	Yes	Synapse, Presynapse	Cytosolic protein guiding KEFRQ-proteins to chaperone-mediated autophagy (CMA)	(Uytterhoeven et al., 2015; Kaushik and Cuervo, 2018; Andres-Alonso et al., 2021)
HOPS complex Vps11 Vps16/Vps33A Vps18 Vps39 Vps41	Q91W86 (VPS11_MOUSE) Q920Q4 (VPS16_MOUSE) Q9D2N9 (VP33A_MOUSE) Q8R307 (VPS18_MOUSE) Q8R5L3 (VPS39_MOUSE) Q5KU39 (VPS41_MOUSE); VAM2	Yes Yes Yes Yes Yes Yes	Yes SV-visitor Yes SV-visitor Yes SV-visitor Yes SV-visitor P2’-fraction P2’-fraction	Yes Yes No Yes No No	Presynapse, AZ Presynapse Presynapse Presynapse Synaptosome Synaptosome	Homotypic fusion and vacuole protein sorting complex; involved in the fusion events of late endosomes and lysosomes.	(Jiang et al., 2014; Dikic and Elazar, 2018; van der Beek et al., 2019)
Huntingtin/Htt	P42859 (HD_MOUSE), Huntington disease protein homolog;	Yes	Yes SV-visitor	Yes	Synapse, Presynapse; SV	Scaffolding adaptor recruited to autophagosomes	(Deng et al., 2017; Stavoe and Holzbaur, 2018; Cason et al., 2021)
JIP1	Q9WVI9 (JIP1_MOUSE); c-Jun-amino-terminal kinase-interacting protein 1; JNK-interacting protein 1; Islet brain 1 [IB-1]	Yes	No	No	Synapse (Axon)	Motor adaptor for autophagosome	(Saudou and Humbert, 2016; Stavoe and Holzbaur, 2018; Hill and Colon-Ramos, 2020; Cason et al., 2021)
JIP3	Q9ESN9 (JIP3_MOUSE); C-Jun-amino-terminal kinase-interacting protein 3; Unc-16; Mapk8ip3	Yes	P2’-fraction	No	Synapse Synaptosome (Axon)	Motor adaptor for autophagosome	(Hill et al., 2019; Hill and Colon-Ramos, 2020; Cason et al., 2021)
LAMP1,	P11438 (LAMP1_MOUSE); Lysosome-associated membrane glycoprotein 1; CD107 antigen-like family member A	Yes	Yes SV-visitor	Yes	Presynapse, SV	Marker for degradative autophagy-lysosomal organelles	(De Leo et al., 2016; Vukoja et al., 2018; Hill and Colon-Ramos, 2020) but see also (Cheng et al., 2015)
LAMP2(A)	P17047 (LAMP2_MOUSE); Lysosome-associated membrane glycoprotein 2; CD107 antigen-like family member B	No	Yes SV-visitor	No	Presynapse, SV	LAMP2A is chiefly involved in CMA	(Wang et al., 2017; Issa et al., 2018; Kaushik and Cuervo, 2018)
LC3/ATG8	Q9CQV6 (MLP3B_MOUSE); Autophagy-related ubiquitin-like modifier LC3 B; Microtubule-associated proteins 1A/1B light chain 3B; Map1lc3b.	Yes	Yes SV-visitor	Yes	Synapse, Presynapse	Lipidated ATG8-like proteins that are key factors for various autophagic processes; LGG in *C. elegans*;	(Dikic and Elazar, 2018; Hill and Colon-Ramos, 2020; Martens and Fracchiolla, 2020)
LRRK1	Q3UHC2 (LRRK1_MOUSE); Leucine-rich repeat serine/threonine-protein kinase 1	No	No	No	–	Regulates autophagy via TBC1D2-dependent Rab7 inactivation	(Toyofuku et al., 2015)
LRRK2	Q5S006 (LRRK2_MOUSE); Leucine-rich repeat serine/threonine-protein kinase 2; Dardarin	Yes	No	Yes	Synapse, Presynapse	LRRK2 acts on key actors of the SV cycle; among them endophilin A, a main anchor for autophagic proteins	(Soukup et al., 2016, 2018; Azarnia Tehran et al., 2018; Taylor and Alessi, 2020; Piccoli and Volta, 2021)
mTOR	Q9JLN9 (MTOR_MOUSE); Serine/threonine-protein kinase mTOR; mechanistic target of rapamycin;	Yes	Yes SV-visitor	Yes	Synapse, Postsynapse SV fraction	Regulates various cellular processes including autophagy	(Bockaert and Marin, 2015)
MYCBP2	Q7TPH6 (MYCB2_MOUSE); E3 ubiquitin-protein ligase MYCBP2; Pam/highwire/rpm-1 protein	Yes	Yes SV-visitor	No	Presynapse SV fraction	E3 ligase upstream of ULK1/Unc51	(Grill et al., 2016; Crawley et al., 2019)
NBR1	P97432 (NBR1_MOUSE); Next to BRCA1 gene 1 protein	No	No	No	–	Autophagy receptor	(Kirkin et al., 2009; Deng et al., 2017; Dikic and Elazar, 2018)
NDP52	A2A6M5 (CACO2_MOUSE); Calcium-binding and coiled-coil domain-containing protein 2; Nuclear domain 10 protein 52	No	No	No	–	Autophagy receptor	(Deng et al., 2017; Dikic and Elazar, 2018; Chang et al., 2021)
NRBF2	Q8VCQ3 (NRBF2_MOUSE) Nuclear receptor-binding factor 2	No	No	No	–	Assembles with PI3KC3, Autophagosome maturation, Rab7 effector	(Cai et al., 2021)
OPTN	Q8K3K8 (OPTN_MOUSE) Optineurin	No	No	No	–	Autophagy receptor	(Deng et al., 2017; Dikic and Elazar, 2018; Chang et al., 2021)
OCRL	Q6NVF0 (OCRL_MOUSE) Inositol polyphosphate 5-phosphatase OCRL	No	Yes SV-visitor	No	Presynapse SV fraction	Lipid phosphatase, controls autophagosome-lysosome fusion	(De Leo et al., 2016; Marat and Haucke, 2016; Palamiuc et al., 2020)
Parkin	Q9WVS6 (PRKN_MOUSE); E3 ubiquitin-protein ligase parkin	Yes	No	Yes	Synapse, Presynapse, SV	E3 ubiquitin-protein ligase involved in mitophagy and together with Bassoon controls SV protein degradation.	(Mouatt-Prigent et al., 2004; Dikic and Elazar, 2018; Soukup et al., 2018; Hoffmann-Conaway et al., 2020)
p62/SQSTM1	Q64337 (SQSTM_MOUSE) Sequestosome-1, Ubiquitin-binding protein p62	No	P2’-fraction	No	Synapse, Synaptosome Presynapse	Autophagy receptor Presynaptic localization in primary neurons.	(Deng et al., 2017; Dikic and Elazar, 2018; Soukup et al., 2018) (Okerlund et al., 2017)
p115/Uso1	Q9Z1Z0 (USO1_MOUSE), General vesicular transport factor p115; Protein USO1 homolog; Transcytosis-associated protein	Yes	P2’-fraction	No	Synapse, Synaptosome	Associates with PI3KC3complex I	(Dikic and Elazar, 2018)
PHLPP1	Q8CHE4 (PHLP1_MOUSE), Pleckstrin homology domain leucine-rich repeat-containing protein phosphatase 1; PH domain-containing family E member 1	No	No	No	n.d.	Phosphatase in CMA; dephosphorylates e.g., AKT1	(Arias et al., 2015; Kaushik and Cuervo, 2018)
PLEKHG5	Q66T02 (PKHG5_MOUSE) Pleckstrin homology domain-containing family G member 5; Synectin-binding RhoA exchange factor; Tech	Yes	No	(Yes) only BP	Presynapse	Regulates autophagy of SV; Guanine exchange factor (GEF) that regulates the activity of Rab26.	(Lüningschrör et al., 2017; Hill and Colon-Ramos, 2020; Andres-Alonso et al., 2021)
PLEKHM1	Q7TSI1 (PKHM1_MOUSE); Pleckstrin homology domain-containing family M member 1	No	No	No	n.d.	Rab7 and Arl8 effector; recruits HOPS complex to autophagosome	(Hill and Colon-Ramos, 2020; Andres-Alonso et al., 2021; Borchers et al., 2021)
Rab7	P51150 (RAB7A_MOUSE), Ras-related protein Rab-7a	Yes	Yes SV-Visitor	Yes	Presynapse, SV	Small GTPase with key role for the maturation of late endosomes and autophagosomes	(Hyttinen et al., 2013; Weingarten et al., 2014; Hill and Colon-Ramos, 2020; Borchers et al., 2021; Xing et al., 2021)
Rab11a	P62492 (RB11A_MOUSE); Ras-related protein Rab-11A	No	Yes SV-visitor	Yes	Postsynapse Presynapse SV	Rab of recycling endosomes (RE); involved in phagophore formation from RE	(Binotti et al., 2016; Puri et al., 2018; Wei and Duan, 2019)
Rab12	P35283 (RAB12_MOUSE); Ras-related protein Rab-12	No	Yes SV-visitor	No	Presynapse, SV	Rab involved in autophagy initiation; LRRK2 substrate	(Takamori et al., 2006; Matsui and Fukuda, 2013; Wei and Duan, 2019; Taoufiq et al., 2020)
Rab24	P35290 (RAB24_MOUSE); Ras-related protein Rab-24; Rab-16;	Yes	Yes SV-visitor	No	Synapse Presynapse SV	Facilitates clearance of autophagic compartments;	(Yla-Anttila et al., 2015; Wei and Duan, 2019)
Rab26	Q504M8 (RAB26_MOUSE); Ras-related protein Rab26	Yes	Yes SV-resident	Yes	Synapse Presynapse, SV	Links SV to autophagy pathway; in complex with Plekhg5;	(Binotti et al., 2015; Lüningschrör et al., 2017; Wang et al., 2017; Andres-Alonso et al., 2021; Kohrs et al., 2021)
Rab35	Q6PHN9 (RAB35_MOUSE); Ras-related protein Rab-35	Yes	Yes SV-visitor	Yes	Synapse, Presynapse, SV	Small GTPase controlling SV turnover; acts via NDP52	(Sheehan et al., 2016; Minowa-Nozawa et al., 2017; Soukup et al., 2018; Wei and Duan, 2019)
Rab37	Q9JKM7 (RAB37_MOUSE); Ras-related protein Rab-37	Yes	Yes SV-visitor	No	Synapse Presynapse SV	Interacts with ATG5 and regulates ATG5-12-16 complex assembly	(Sheng et al., 2018; Wei and Duan, 2019)
Rab39a	Q8BHD0 (RB39A_MOUSE); Ras-related protein Rab-39A	Yes	Yes SV-visitor	No	Presynapse SV	Interacts with PIK3C3 and negatively regulates autophagosome formation;	(Behrends et al., 2010; Boyken et al., 2013; Seto et al., 2013)
RHEB	Q921J2 (RHEB_MOUSE); GTP-binding protein Rheb; Ras homolog enriched in brain	Yes	Yes SV-visitor	Yes	Postsynapse Preynapse SV	Small GTPase in mTORC1 signaling pathway	(Bockaert and Marin, 2015)
RTN3	Q9ES97 (RTN3_MOUSE); Reticulon-3;	Yes	Yes SV-visitor	Yes	Presynapse SV	ER protein involved in ER-phagy	(Weingarten et al., 2014; Grumati et al., 2018; Beese et al., 2019; Stavoe and Holzbaur, 2019; Kuijpers et al., 2021; Wojnacki et al., 2021)
RUSC2	Q80U22 (RUSC2_MOUSE); RUN and SH3 domain-containing protein 2; Iporin	No	No	No	n.d.	Regulates association of ATG9a with kinesin motor	(Guardia et al., 2021)
SIPA1L2	Q80TE4 (SI1L2_MOUSE); Signal-induced proliferation-associated 1-like protein 2; SPAR2	Yes	P2’-fraction	No	Synaptosome Presynapse	Rap-GTPase activating protein (RapGAP); component of signaling amphisome; co-traffics with Snapin; colocalized with synaptophysin in presynapses.	(Andres-Alonso et al., 2019, 2021)
SNAP29	Q9ERB0 (SNP29_MOUSE); Synaptosomal-associated protein 29,	Yes	Yes SV-resident	Yes	Presynapse, SV	Autophagosome fusion with endolysosome	(Itakura et al., 2012; Diao et al., 2015; Andres-Alonso et al., 2021)
Snapin	Q9Z266 (SNAPN_MOUSE); SNARE-associated protein Snapin; Biogenesis of lysosome-related organelles complex 1 subunit 7; BLOC-1S7.	Yes	P2’-fraction	Yes	Synaptosome Presynapse	Motor adaptor that coordinates retrograde transport and late endosomal-lysosomal trafficking	(Cai et al., 2010; Kuijpers et al., 2020; Andres-Alonso et al., 2021)
SNX4	Q91YJ2 (SNX4_MOUSE); Sorting nexin-4; ATG24	Yes	(Yes) P2’-fraction	Yes	Synaptosome Presynaptic endosome SV	Phosphatidylinositol 3-phosphate-binding protein that controls ATG9A recycling and autophagy	(Ravussin et al., 2021)
Synaptojanins	Q8CHC4 (SYNJ1_MOUSE); Q9D2G5 (SYNJ2_MOUSE); Synaptojanin-1, -2; Synaptic inositol 1,4,5-trisphosphate 5-phosphatase 1, 2.	Yes (both isoforms)	Yes (Synj1) SV-visitor	Yes (both isoforms)	Presynapse SV fraction	Lipid phosphatase that is essential for maturation of autophagosmes in presynaptic boutons	(Vanhauwaert et al., 2017; Azarnia Tehran et al., 2018; Soukup et al., 2018)
Synaptopodin	Q8CC35 (SYNPO_MOUSE);	Yes	Yes SV-visitor	Yes	Postsynapse Presynapse? SV	In cooperation with BAG3 affects fusion between autophagosomes and lysosomes.	(Ji et al., 2019; Taoufiq et al., 2020)
Stx17	Q9D0I4 (STX17_MOUSE); Syntaxin-17;	No	Yes SV-visitor	No	SV fraction	Autophagosome fusion with endolysosome	(Itakura et al., 2012; Jiang et al., 2014; Diao et al., 2015; Andres-Alonso et al., 2021)
Tax1bp1	Q3UKC1 (TAXB1_MOUSE); Tax1-binding protein 1 homolog	No	P2’-fraction	No	Synaptosome	Autophagy receptor; Ubiquitin-binding protein that mediates autophagosome induction	(Whang et al., 2017; Chang et al., 2021)
TBC1D24/ Skywalker	Q3UUG6 (TBC24_MOUSE); TBC1 domain family member 24; Q9VIH7 (SKY_DROME); GTPase-activating protein skywalker	Yes	P2’-fraction	Yes	Synapse Presynapse	GTPase activating protein controlling SV turnover; acts on Rab35; regulates autophagy via TRAPP complex and ATG9	(Fernandes et al., 2014; Lamb et al., 2016; Soukup et al., 2018; Soykan et al., 2021)
TBC1D2	B1AVH7 (TBD2A_MOUSE); TBC1 domain family member 2A; Armus	No	No	(Yes) only BP	Synapse	GTPase-activating protein for RAB7A	(Toyofuku et al., 2015; Jaber et al., 2016)
TBK1	Q9WUN2 (TBK1_MOUSE); Serine/threonine-protein kinase TBK1; TANK-binding kinase 1	Yes	P2’-fraction	No	Synapse	Regulates together with Rab35 NDP52 recruitment to promote mitophagy and maturation of autophagosomes.	(Minowa-Nozawa et al., 2017)
Tecpr1	Q80VP0 (TCPR1_MOUSE) Tectonin beta-propeller repeat-containing protein 1	No	Yes SV-resident	No	Presynapse SV	Autophagosome maturation mediated by TECPR1 and the ATG12-ATG5 conjugate;	(Chen et al., 2012; Wetzel et al., 2020)
TRAPP complex TRAPPC8	Q9Y2L5 (TPPC8_HUMAN); Trafficking protein particle complex 8	No	Yes SV-resident	No	Presynapse SV	TRAPPC8 is the mammalian ortholog a yeast autophagy-specific TRAPP subunit. It interacts with TBC1D24 to regulate ATG9 trafficking;	(Lamb et al., 2016)
Tsc2	Q61037 (TSC2_MOUSE); Tuberin; Tuberous sclerosis 2 protein homolog	Yes	P2’-fraction	Yes	Synapse Synaptosome Postsynapse	Controls mTORC1 signaling; TSC2 is regulated by WIPI3 and FIP200; heterozygous loss of TSC2 function impairs spine development.	(Tang et al., 2014; Bakula et al., 2017; Dikic and Elazar, 2018; Tomoda et al., 2020)
Ubqln2	Q9QZM0 (UBQL2_MOUSE) Ubiquilin-2; Chap1; DSK2 homolog; PLIC-2	No	P2’-fraction	No	Synaptosome	Ubiquitn binding autophagy receptor	(Deng et al., 2017; Lin et al., 2021)
ULKs/ATG1-like ULK1 ULK2 ULK3	O70405 (ULK1_MOUSE); Unc51-like kinase 1; Serine/threonine-protein kinase ULK1//Q9QY01 (ULK2_MOUSE); Unc-51-like kinase 2	No	(Yes) ULK3 P2’-fraction	No	Synaptosome (SV)	Initiation of autophagy; ULK2 has important role for excitation-inhibition balance in the brain	(Lee and Tournier, 2011; Alers et al., 2012; Dikic and Elazar, 2018; Sumitomo et al., 2018; Tomoda et al., 2020; Chang et al., 2021)
Uvrag	Q8K245 (UVRAG_MOUSE); UV radiation resistance-associated protein	No	Yes SV-visitor	No	Presynapse SV	Regulatory component of PIK3C2; involved in maturation of autophagosomes;	(Mercer and Tooze, 2021)
VAMP7	P70280 (VAMP7_MOUSE); Vesicle-associated membrane protein 7; Synaptobrevin-like protein 1; TI-VAMP	Yes	Yes SV-resident	Yes	Presynapse SV	Overlapping functions with VAMP8, SNARE of secretory lysosomes in astrocytes.	(Verderio et al., 2012; Toyofuku et al., 2015; Hill and Colon-Ramos, 2020; Tian et al., 2021; Wojnacki et al., 2021)
VAMP8	O70404 (VAMP8_MOUSE); Vesicle-associated membrane protein 8	No	No	No	n.d.	SNARE involved in autophagosome fusion with endolysosome together with STX17 and SNAP29	(Itakura et al., 2012; Diao et al., 2015; Andres-Alonso et al., 2021; Tian et al., 2021)
Vps13 (a,c,d)	Q5H8C4 (VP13A_MOUSE); Vacuolar protein sorting-associated protein 13A	No	Yes (13d) P2’-fraction (13a,c)	No	Synaptosome Presynapse SV	ATG2-like protein involved in ER-phagy.	(Chen et al., 2020; Chang et al., 2021)
Vps15/PIK3R4	Q8VD65 (PI3R4_MOUSE); Phosphoinositide 3-kinase regulatory subunit 4;	No	Yes SV-visitor	No	Presynapse SV	Regulatory subunit in the PI3KC3 complex;	(Mercer and Tooze, 2021)
Vps33B	P59016 (VP33B_MOUSE); Vacuolar protein sorting-associated protein 33B	No	Yes SV-visitor	Yes	Presynapse, SV	belongs to class C core vacuole/endosome tethering (CORVET) complex, which is mainly implicated in endosomal fusion	(Takamori et al., 2006; Jiang et al., 2014; van der Beek et al., 2019)
Vps34/PIK3C3	Q6PF93 (PK3C3_MOUSE); Phosphatidylinositol 3-kinase catalytic subunit type 3; Vps34	No	Yes SV-resident	No	Presynapse SV	Catalytic component of PIK3C3–C1; colocalizes with synaptophysin at synapses.	(Inaguma et al., 2016; Jaber et al., 2016; Dikic and Elazar, 2018)
Vps35	Q9EQH3 (VPS35_MOUSE); Vacuolar protein sorting-associated protein 35; Vesicle protein sorting 35	Yes	P2’-fraction	Yes	Synapse Synaptosome Presynapse, Peri-AZ	Component of the retromer complex; involved in SV endocytosis in cooperation with LRRK2. Knock-down causes accumulation of ATG9a on endolysosomes	(Inoshita et al., 2017; Kaushik and Cuervo, 2018; Ravussin et al., 2021)
WDFY3/ALFY	Q6VNB8 (WDFY3_MOUSE), WD repeat and FYVE domain-containing protein 3, Autophagy-linked FYVE protein	No	Yes SV-visitor	No	Presynapse SV	Autophagy receptor; mainly for aggrephagy,	(Deng et al., 2017; Stavoe and Holzbaur, 2019)
WDR47	Q8CGF6 (WDR47_MOUSE); WD repeat-containing protein 47; Neuronal enriched MAP interacting protein	Yes	P2’-fraction	No	Synapse Synaptosome	Negatively regulates association of ATG9a with kinesin motor; essential for autophagy	(Kannan et al., 2017; Tomoda et al., 2020; Guardia et al., 2021)
WDR91	Q7TMQ7 (WDR91_MOUSE); WD repeat-containing protein 91	Yes	No	No	Synapse	Rab7 effector, regulates lysosome fusion.	(Borchers et al., 2021; Xing et al., 2021)
WIPI2/ATG18a	Q80W47 (WIPI2_MOUSE); WD repeat domain phosphoinositide-interacting protein 2	No	P2’-fraction	No	Synapse, Synaptosome	Involved in early steps of phagophore formation, recruits ATG12-ATG5-ATG16L1 E3-like complex.	(Vanhauwaert et al., 2017; Dikic and Elazar, 2018; Stavoe et al., 2019)
WIPI3/WDR45B WIPI4/WDR45	Q9CR39 (WIPI3_MOUSE) Q91VM3 (WIPI4_MOUSE); WD repeat domain phosphoinositide-interacting protein 3/4; WD repeat-containing protein 45/45B	No	P2’-fraction	No	Synaptosome	Components of the autophagy machinery	(Bakula et al., 2017; Wan et al., 2020)

*AZ, active zone; BP, annotated for biological processes; CMA, chaperone-mediated autophagy; SV, synaptic vesicle; n.d., not detected in synapses; P2’, Synaptosomal Protein Preparation (Taoufiq et al., 2020).*

*^a^www.synprot.de (Pielot et al., 2012).*

*^b^As reported by Taoufiq et al. (2020).*

*^c^www.syngoportal.org (Koopmans et al., 2019).*

*^d^As taken from the databases or as referred to in remarks/function column.*

## Bassoon, A Docking Station for Protein Turnover Pathways at the Active Zone

The AZ of neurotransmitter release is characterized by a prominent electron-dense meshwork of proteins, the CAZ, that organizes the regulated fusion of SVs with the presynaptic cell membrane (Garner et al., 2000; Gundelfinger and Fejtova, 2012; Sudhof, 2012; Ackermann et al., 2015). Two related multidomain proteins, Bassoon and Piccolo, serve among others as major scaffolding proteins of the CAZ (Gundelfinger et al., 2016). Knock-down of these two proteins has severe effects on the maintenance and integrity of neuronal synapses, which is in part mediated by their interaction with the E3 ubiquitin ligase Siah1 (Waites et al., 2013). More recent studies have shown that while Piccolo has important functions in SV retrieval (Ackermann et al., 2019), Bassoon contributes important binding sites for components of the proteosomal and the autophagic proteostasis pathways. Thus, Bassoon binds, in addition to Siah1, also the proteasomal subunit PSMB4 (alias β7) and thereby controls proteasome assembly (Montenegro-Venegas et al., 2021) as well as the autophagy protein ATG5, part of an E3-like protein ligase, and thus negatively regulates presynaptic autophagy (Okerlund et al., 2017). As will be detailed below, knockout of Bassoon causes increased ubiquitination of various presynaptic proteins, including various SV proteins, and enhances presynaptic autophagy (Hoffmann-Conaway et al., 2020) as well as proteasome activity (Montenegro-Venegas et al., 2021). Interestingly, the E3 ubiquitin ligase Parkin, an enzyme that is also involved in mitophagy and has been implicated in early onset Parkinsonism (Kitada et al., 1998; Dikic and Elazar, 2018), seems to antagonize Bassoon in this function (Montenegro-Venegas et al., 2020). How exactly Parkin acts in this context is unclear, as to date no physical interaction between Parkin and Bassoon has been detected. Interestingly, Parkin ubiquitinates constituents of the endocytic systems in the presynapse, including Synaptojanin-1, Endophilin-A and Dynamin-1 (Cao et al., 2014; Soukup et al., 2018). This could set the framework for the search for relevant functional interactions between Bassoon and the endocytic machinery to control induction of autophagy in the presynapse.

Overall, the findings discussed above suggest that Bassoon is a negative regulator of presynaptic autophagy and the UPS in the presynapse, and as such may act as an anchoring and control point of these two protein turnover pathways. It should be noted, in this context, that Bassoon itself, in contrast to Piccolo, is subject to autophagic degradation upon nutrient limitation in primary neuronal cultures (Catanese et al., 2018). This suggests that Bassoon may be highly feed-back regulated by the processes that it controls.

Interestingly, in *C. elegans*, another active zone protein, Clarinet, was recently reported to regulate presynaptic autophagy (Xuan et al., 2017, 2021). Clarinet is a large AZ protein that occurs in three different isoforms of about 9,000, 3,000, and 1,000 amino acids in length, that is required for proper synapse function. All three isoforms share a PDZ and a C2 domain similar to mammalian Piccolo, and Clarinet’s long and short isoforms are supposed to organize SV recruitment and clustering at the presynaptic AZ (Xuan et al., 2017). An elegant follow-up study by the same lab, uploaded recently onto the bioRxiv server, demonstrates that the long Clarinet isoform controls presynaptic autophagy by regulating ATG9 trafficking at the peri-active endocytic zone (Xuan et al., 2021). These observations further support the concept that large scaffolding proteins of presynaptic AZs couple exo- and endocytic zones at neurotransmitter release sites (Gundelfinger et al., 2003; Haucke et al., 2011) and can have essential roles in organizing presynaptic autophagy processes.

## Constituents of the Endocytic Machinery Associated With Presynaptic Autophagy

A tight linkage between autophagic and endocytic factors has been suggested previously (Soukup and Verstreken, 2017; Vijayan and Verstreken, 2017; Azarnia Tehran et al., 2018). Here, we discuss proteins involved in endocytic processes in neurons that have also been implicated in membranous organelle-based protein turnover as listed in Table 1. Endophilin-A/EndoA, a BAR-domain protein that is able to sense and modify membrane curvature, is crucially involved in Clathrin-dependent and -independent retrieval of membranes following the fusion SVs with the presynaptic AZ (Milosevic et al., 2011; Boucrot et al., 2015; Watanabe et al., 2018). More recent studies also suggest a function for Endophilins A in exocytosis of neurosecretory vesicles (Gowrisankaran et al., 2020) and the coordination of exo- and endocytic processes in presynapses (Kroll et al., 2019). Compensatory endocytosis associated with neurotransmitter release occurs primarily next to the AZ at the so-called peri-AZ (Haucke et al., 2011; Cano and Tabares, 2016). Two studies, one in *Drosophila* (Soukup et al., 2016) and the other one in mice (Murdoch et al., 2016) have identified Endophilin-A also as major player in generating initiation sites for autophagy. The former study showed that phosphorylation of the BAR domain of Endophilin-A by the leucine-rich repeat S/T protein kinase LRRK2 leads to membrane deformation putatively opening entry sites for the autophagy-related protein ATG3. ATG3 in turn can conjugate LC3/ATG8 to phosphatidyl-ethanolamine in the membrane, promoting progression of autophagosome formation (Soukup and Verstreken, 2017). Utilizing mutants for all three EndoA genes in the mouse genome, the second study revealed that partial or complete Endophilin-A-deficiency leads to age-dependent neurodegenerative changes in the brain and up-regulation of the E3 ubiquitin ligase FBXO32. Endophilins-A are essential for autophagosome formation and their proper interplay with FBXO32 coordinates the balance between autophagosomal and UPS-mediated protein turnover and maintains neuronal health (Murdoch et al., 2016). However, to date, FBXO32 has not yet been detected in the presynapse.

Synaptojanins are lipid phosphatases acting on phosphatidylinositols. In particular, the brain-enriched isoform Synaptojanin-1 is recruited to Endophilin-A complexes and, in the SV cycle, is required for Clathrin uncoating after endocytosis (Cremona et al., 1999; Verstreken et al., 2003). More recently, studies on the *Drosophila* larval neuromuscular junction revealed that an inactivating mutation in the SAC1 domain, one of the two enzymatically active lipid phosphatase domains of Synaptojanin, causes accumulation of lipid-binding protein ATG18a on nascent autophagosomes (Vanhauwaert et al., 2017). Interestingly, this mutation does not interfere with SV cycling. Similarly, accumulation of WIPI2, the mammalian ortholog of ATG18a, occurs in neurons derived from induced pluripotent stem cells from a human patient with the same mutation (Vanhauwaert et al., 2017).

The heterooligomeric AP-2 complex is also involved in Clathrin-mediated endocytosis during SV recycling, where it can act at the cell membrane or at a bulk-endocytosed membrane compartment (McMahon and Boucrot, 2011; Kononenko et al., 2014). In addition, AP-2 in cooperation with CALM/PICALM has been implicated in autophagic degradation of the C-terminal fragment of the amyloid precursor protein (APP), thus contributing to the clearance of the Alzheimer-related Aβ peptide (Tian et al., 2013). APP is a cell adhesion molecule that has been detected as a constituent of the presynaptic AZ (Lassek et al., 2013). Moreover, AP-2 can directly interact with ATG9A, a multispan transmembrane protein delivering membrane material for phagophore formation and extension (Imai et al., 2016; Dikic and Elazar, 2018). This interaction appears essential for the trafficking of ATG9A through the recycling endosome and making it available for the autophagy process (Imai et al., 2016). Similarly, delivery of ATG16L1 and in turn ATG12-ATG5 to the phagophore depends on Clathrin and AP-2 (Ravikumar et al., 2010), indicating a key role for AP-2 in autophagy initiation and progression.

Recent studies have revealed that AP-2, in conjunction with the RapGTPase-activating protein (RapGAP) SIPA1L2, serve unexpected roles in the transport of autophagosomes that contain actively signaling BDNF-activated TrkB receptors (Kononenko et al., 2017; Andres-Alonso et al., 2019). AP2 and SIPA1L2 link the TrkB receptor to a Dynein motor for retrograde trafficking via a direct interaction with Snapin, a component of the BLOC-1 complex (see below). Interestingly, SIPA1L2 concurrently associates via LC3 to Rab7-positive amphisomes and binding to LC3 promotes RapGAP activity. Endosomes fuse with autophagosomes to form amphisomes, and this step is required for the degradation of some proteins and the overall function of autophagy and the endosomal system (Cheng et al., 2015). Amphisomes are transient intermediate organelles that in non-neuronal cells rapidly enter a lysosomal-degradative pathway. However, in distal axons mature lysosomes are rare and therefore TrkB-LC3-SIPA1L2-AP2-carrying amphisomes have a longer lifespan. They thus can traffic retrogradely along axons and visit presynaptic boutons. Intriguingly, motility and signaling of amphisomes are controlled by SIPA1L2, whose RapGAP activity reduces the trafficking velocity near boutons (Andres-Alonso et al., 2019). Collectively, these data suggest that retrograde transport of BDNF/TrkB in neuronal amphisomes is involved in plasticity-relevant local signaling at presynaptic boutons and this process seems tightly coupled to autophagy.

LRRK2 is a crucial enzyme with roles in intracellular membrane trafficking, including functions in the SV cycle, autophagy and the endolysosomal system (Taylor and Alessi, 2020; Piccoli and Volta, 2021). VPS35 is the cargo binding component of the retromer complex, which can serve multiple functions in synapses (Brodin and Shupliakov, 2018). VPS35 is an upstream regulator of LRRK2 and, as LRRK2, it is linked to various neurodegenerative diseases including Parkinson’s disease. Inoshita et al. (2017) studied the functional interaction of these two proteins at the *Drosophila* larval neuromuscular junction. Here, they could be localized peripherally to the AZ and inside presynaptic boutons, where they are essential for proper SV retrieval (Inoshita et al., 2017). VPS35 constructs carrying a Parkinson’s disease-associated mutation were unable to complement this endocytotic function in VPS35 null mutants. Tight interaction of the Rab7-LRRK2 pathway and the retromer complex was also observed in fly and rat neurons, where overexpression of wildtype, but not mutant, VPS35 protein was able to rescue Parkinson-related sorting defects (MacLeod et al., 2013).

Proteins discussed in this section, i.e., Endophilin-A, Synaptojanin, AP-2, CALM/PICALM, LRRK2 and VPS35, are all associated with endocytic functions within the SV cycle (cf. Table 1). Evidently, they also contribute to the delivery of key components into membrane-based protein degradation processes. However, the spatial organization of this latter machinery and the question of where within the boutons phagophore assembly is initiated remains to be clarified. Similarly, the crosstalk between SV recycling and autophagic and endocytic pathways needs further attention (see also Overhoff et al., 2021).

## Components of Membrane-Based Protein Turnover Pathways Associated With Synaptic Vesicles

**FIGURE 3:**
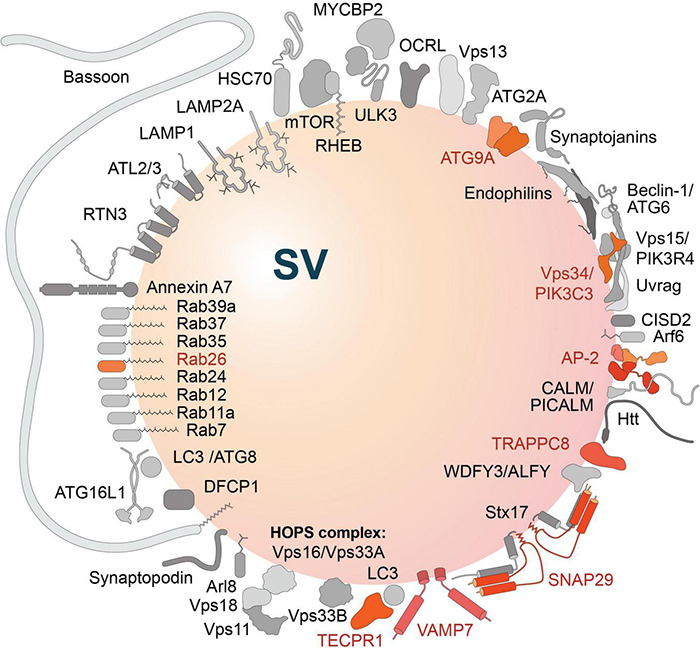
Autophagy-related proteins detected in the hidden proteome of synaptic vesicles (SV) (Taoufiq et al., 2020). Proteins of the SV-resident repertoire are indicated in different shades of orange; proteins defined by the authors as SV-visitors are gray-shaded. Note, the large active zone scaffolding protein Bassoon may be anchored to SVs via N-terminal myristoylation (Dresbach et al., 2003).

A link between SV cycling and membrane trafficking processes controlling proteostasis is also indicated by the finding that constituents of autophagy and endolysosomal degradation pathways are associated with SV protein preparations (Takamori et al., 2006; Boyken et al., 2013; Taoufiq et al., 2020). These are also listed in Table 1 and include, e.g., small GTPases like Rab7, Rab11a, Rab12, Rab24, Rab26, Rab35, Rab37, Rab39a, Arf6 and the ADP-ribosylation factor-like GTPase Arl8, the SNARE proteins SNAP-29, Stx17 and VAMP7, constituents of the phosphatidylinositol 3-kinase class III (PIK3C3) complex (Vps34, Vps15, Beclin-1, CISD2, Uvrag), as well as other autophagy-related proteins, i.e., ATG2a, ATG9A, ATG16L1, LC3/ATG8, Tecpr1, and autophagy the receptors WDFY3/ALFY, components of the TRAPP complex and various others. Altogether, Taoufiq et al. (2020) identified ∼40 autophagy-related proteins within the hidden SV proteome, eleven of them in the SV-resident repertoire, while the others were defined as SV-visitors by the authors (Figure 3).

Of particular interest is the presence of ATG9 in SVs (Boyken et al., 2013; Taoufiq et al., 2020), a lipid scramblase that is involved in early steps of phagophore formation (Rao et al., 2016; Guardia et al., 2020; Maeda et al., 2020; Matoba and Noda, 2020; Matoba et al., 2020; Orii et al., 2021). Here, it may play a central role in organizing presynaptic autophagy in cooperation with the endocytic machinery (see below).

Rab26 is associated with a subset of recycled SVs, directing them for autophagic degradation (Binotti et al., 2015). This is modulated by the Pleckstrin homology containing family member 5 (PLEKHG5), which acts as guanine exchange factor (GEF) for Rab26. In motoneurons, the lack of PLEKHG5 leads to defective axon growth and impaired SV autophagy, a mechanism that may underlie motoneuron disease (Lüningschrör et al., 2017; Lüningschrör and Sendtner, 2018).

The presence of Arl8 in SV preparations is interesting as this protein has been characterized as a factor associated with anterogradely transported degradative lysosomes (Farias et al., 2017; Farfel-Becker et al., 2019). Vukoja et al. (2018) have proposed that SV precursors may share lysosomal membrane markers, but are distinct from bona fide lysosomes. Another, more recent study reported that Arl8 is involved exclusively in the anterograde transport of lysosomes (De Pace et al., 2020). In addition, Arl8 is involved in controlling fusion of autophagosomes with lysosomes (see Lieberman and Sulzer, 2020), where it may contribute to SV turnover via axonal autolysosomes.

SNAP-29 is regarded as an integral protein of vesicular membranes in the molecular SV model (Takamori et al., 2006; Taoufiq et al., 2020). In addition, SNAP-29, together with VAMP8, are considered as late endosomal/lysosomal SNAREs involved in the fusion with autophagosomes upon forming a SNARE complex with autophagosomal SNARE Syntaxin 17 (Itakura et al., 2012; Hill and Colon-Ramos, 2020).

In addition, multiple constituents of the endocytic machinery and the AZ, such as AP-2 complex, Bassoon, CALM, Endophilin-A, Endophilin-B1, Synaptojanin and VPS35, are associated with SV preparations (Table 1). Further proteins detected in SV preparations include the lysosomal-associated membrane protein LAMP1, the E3-ubiquitin ligase Parkin, Vps33B, a protein associated with late endosomes, as well as the Annexin A7. These proteins are mainly considered as visitor proteins associated with a subset of SVs (Figure 3). In many cases, however, their (pre-)synaptic localization has been reported utilizing immunocytochemistry.

A number of additional proteins with known functions in autophagic and/or endolysosomal degradative pathways could be localized to presynaptic structures as extracted from published literature (Table 1; SynGO database). Among them are:

- Atlastin2/3, which are involved in initiation of phagophore formation by guiding the Unc51-like kinase (ULK1) complex to the ER (Liu et al., 2021).
- Multiple GABARAPs that, as LC3, belong to the family of ATG8-like proteins that can be conjugated to phosphatidylethanolamine and are required for phagophore extension and closure (e.g., Martens and Fracchiolla, 2020).
- ATG5 that together with ATG12 and ATG16L forms an E3-like complex for the conjugation of ATG8/LC3 family members to phosphatidylethanolamine (Chang et al., 2021).
- Vps34, the catalytic kinase subunit of the lipid kinase PIK3C3-Complex 1, acting in phagophore nucleation (Dikic and Elazar, 2018; Chang et al., 2021).
- Sorting nexin 4 (SNX4)/ATG24B, a PI3P-binding protein controlling the recycling of ATG9A for reuse in phagophore formation (Ravussin et al., 2021).
- The cargo adaptors p62/SQSTM1 and Huntingtin (Deng et al., 2017).
- Snapin, a subunit of the BLOC-1 complex, which functions as a motor adaptor that coordinates retrograde transport and late endosomal-lysosomal trafficking (Cai et al., 2010). Moreover, Dynein-Snapin-mediated retrograde transport was reported to promote clearance of presynaptic mitophagosomes (Han et al., 2020).
- TBC1D24/skywalker, a GTPase-activating protein acting on Rab35 in the endolysosomal pathway (Fernandes et al., 2014; Wang et al., 2017) as well as on the small GTPase Arf6, which antagonizes Rab35 in SV recycling from early endosomes (Sheehan et al., 2016; Sheehan and Waites, 2019).
- Arf6, which in addition has been reported as a regulator of autophagosome formation by controlling phosphatidylinositol 4,5-bisphosphate (PIP2) generation and in turn phospholipase D activity (Moreau et al., 2012) and to rescue aberrant autophagosome formation in Synaptojanin-1-deficient in zebrafish cone photoreceptors (George et al., 2016). This includes Arf6 into the list of players in endocytosis-associated initiation of autophagy.
- Components of the HOPS (Homotypic fusion and vacuole protein sorting) complex involved in the fusion of late endosomal and lysosomal compartments (Jiang et al., 2014; van der Beek et al., 2019).
- Reticulon-3 (RTN3), which is involved in ER-phagy (Beese et al., 2019; Stavoe et al., 2019).
- HSC70, a cytosolic chaperonin of the HSP70 family that is involved in microautophagy and chaperone-mediated autophagy (CMA) (Kaushik and Cuervo, 2018), which is known to contribute significantly to the regulation of synaptic protein levels (Uytterhoeven et al., 2015).

Many of the autophagy-related proteins associated with the presynaptic endocytic machinery, the SV proteome, or other presynaptic structures are phospholipid-binding and -modifying proteins. These include, e.g., various subunits of PI3 kinases, PI phosphatases synaptojanin and OCRL, the lipid scramblase ATG9, lipid transfer protein ATG2a, as well as various other phospholipid binding proteins (Table 1). This indicates the significance of the metabolism of phospholipids in general and phosphoinositides in particular for the proper performance of presynaptic autophagy-related processes (for a detailed review see Marat and Haucke, 2016; Palamiuc et al., 2020).

**FIGURE 4:**
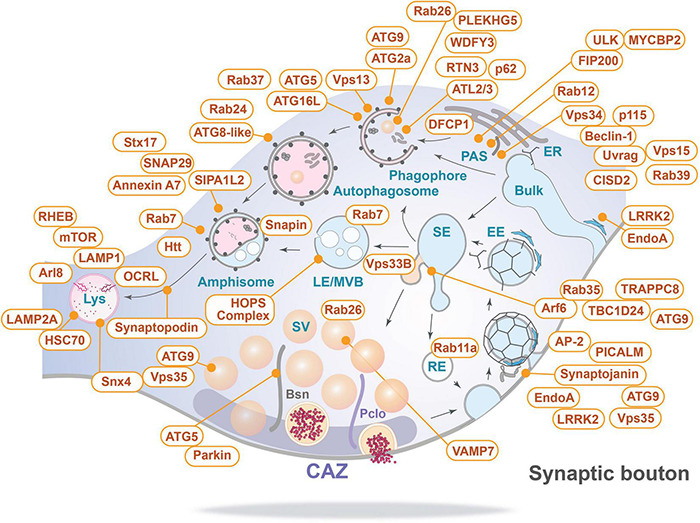
Inventory of autophagy-related proteins detected in presynaptic boutons and their relevant sites of action (for details see text and Table 1). Bsn, Bassoon; CAZ, cytomatrix at the active zone; EE, early endosome; ER, endoplasmic reticulum; LE/MVB, late endosome/multi-vesicular body; Lys, lysosome; PAS, phagophore assembly site/pre-autophagosomal structure; Pclo, Piccolo; RE, recycling endosome; SE, sorting endosome; and Bulk indicates bulk endocytosis.

Based on this inventory of autophagy-related proteins present at the presynapse (Figure 4), we can now start discussing potential cellular mechanisms of membrane-associated protein turnover in this compartment.

## Tagging and Recruitment of Presynaptic Autophagic Cargos

A central feature of autophagic function is the recruitment and degradation of aged and damaged proteins and membranous organelles. As in most forms of autophagy in different cells and organisms, presynaptic boutons seem to use the ubiquitination system to tag proteins not only for proteasomal degradation, but also in many instances for their removal by autophagy. This versatile system is capable of attaching specific ubiquitin chains to substrates, and this ubiquitination coding plays an essential role in recruitment to autophagic structures (Deng et al., 2017; Kwon and Ciechanover, 2017). The process of substrate ubiquitination requires the regulated activation of several key component of this tagging system, including the ubiquitin activating enzyme E1, the ubiquitin conjugating enzymes E2 and the E3 adaptor/ligases (Nandi et al., 2006; Ding and Shen, 2008). While there is only one E1 enzyme in mammalian cells, there are tens of E2s and hundreds of E3s (Li et al., 2008; Kawabe and Stegmuller, 2021). Of these, E3s are posited to provide substrate specificity, while the E2s define the type of poly-ubiquitin chain (Kwon and Ciechanover, 2017). These poly-ubiquitin determinants are then recognized by a variety of adaptor proteins that mediate the recruitment of tagged cargos into specific degradation pathways. For example, during autophagy the p62/SQSTM1 adaptor protein is capable of recognizing K63-poly-ubiquitined proteins for their engulfment into autophagic organelles (Linares et al., 2013).

**FIGURE 5:**
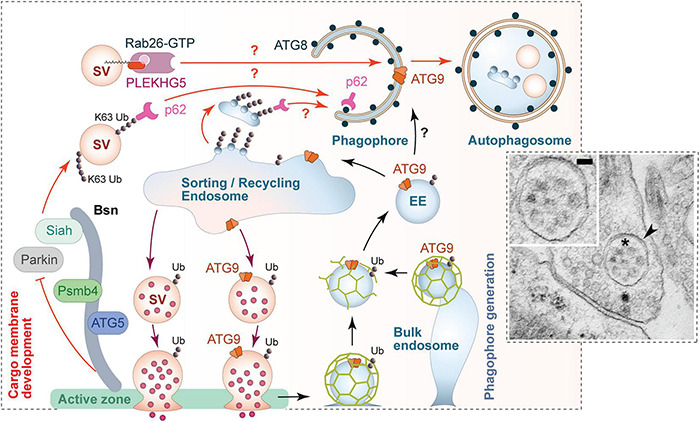
Scenario for the regulation of presynaptic autophagy. Autophagy within presynaptic boutons appears to be locally regulated and mediated via the convergence of two major facets of autophagy. (1) Local tagging of aged and/or damaged proteins/organelles by the ubiquitination system. The active zone protein Bassoon is one regulator of this process by scaffolding E3 ligases such as Siah1 and Parkin. Bassoon can also control the induction of phagophore formation by inhibiting the activity of ATG5 and proteasome function via its binding to Psmb4 proteasomal subunit. (2) The formation of phagophore membranes, which requires an interplay between numerous proteins essential for the regulated conjugation of ATG8/LC3 to membranes containing the integral membrane protein ATG9. Many of these proteins are part of the hidden proteome of SVs (see Figure 3) and thus available for the rapid production of these membranes. This aspect of autophagy seems to be coupled to synaptic activity and the sorting and recycling of SV proteins through early endosomes. While not well understood, this compartment is well positioned to not only sort healthy ensembles of proteins regenerating functional SVs, but also damaged ubiquitinated proteins for engulfment into newly forming phagophores. This latter step requires the small GTPase Rab26 and its guanine exchange factor PLEKHG5, as well as autophagy adaptor proteins, like p62/SQSTM1, which binds both poly-ubiquitin chains and ATG8s. The inserted electron micrograph demonstrates the uptake of entire SV-like structures (asterisk) into autophagic vacuoles (arrowhead) within a presynapse (taken from Hoffmann-Conaway et al., 2020; size bar, 50 nm). Finally, the sorting/recycling endosomes also appears to function in the regeneration of SV-like membranes that carry ATG9, providing autophagic support for boutons in subsequent rounds of neurotransmitter release.

Fundamental questions related to presynaptic autophagy are: which of the many E2 and E3 ligases contribute to presynaptic proteostasis? Further, when are they used and how are they regulated? A number of presynaptic E3 ligases (e.g., Scrapper, FBXO45) have been identified that trigger the removal/destruction of AZ proteins including RIM1, Munc13 or Munc18 via the proteasome (Yao et al., 2007; Tada et al., 2010; Martin et al., 2014). Less is known about which E3s regulate the removal of integral SV proteins. As mentioned above, studies of boutons lacking the presynaptic AZ proteins Piccolo and Bassoon have led to the identification of two E3s, Siah1 and Parkin, linked to the removal of SV proteins via the autophagy degradative system (Waites et al., 2013; Okerlund et al., 2017; Hoffmann-Conaway et al., 2020). Mechanistically, these AZ proteins seem to act as negative regulators of these enzymes. This is best exemplified in studies of Bassoon knockout mice, where the total pool of SVs per bouton is dramatically reduced, which can be restored by knocking down the expression of either Siah1 or Parkin (Hoffmann-Conaway et al., 2020), indicating that Bassoon normally counteracts their actions (Figure 5). For Siah1 this is supported by experiments showing that it directly binds the zinc finger domains in Bassoon, which inhibits Siah1 ubiquitination activity (Waites et al., 2013).

Hints to the substrates of these enzymes come from proteomic studies on synaptosomes isolated from the cortex of Bassoon-deficient mice (Hoffmann-Conaway et al., 2020). These studies identified a dramatic increase in ubiquitinated peptides from SV proteins and proteins involved in SV fusion with the presynaptic membrane, including SNAP25, Synaptotagmin 1, SV2, V-ATPase, VAMP2 and Syntaxin1b. Moreover, autophagy appears to be the major degradative pathway employed, as autophagic vacuoles, but not multi-vesicular bodies, accumulated in these boutons, and inhibiting autophagy restores SV pool size (Hoffmann-Conaway et al., 2020). Intriguingly, this study also found that the Ubiquitin-conjugating enzyme UBE2N was hyper-ubiquitinated. This is highly relevant, as UBE2N is an ubiquitin conjugating enzyme directly involved in creating K63-poly-ubiquitin chains (David et al., 2010), which can tag proteins for degradation via autophagy (Linares et al., 2013). UBE2N can also cooperate with both Siah1 and Parkin (Markson et al., 2009; Fiesel et al., 2014; Geisler et al., 2014). Another E3 ligase, HERC1, was proposed to dysregulate presynaptic pathways and to potentially affect SV autophagy, yet the mode of action is unclear (Montes-Fernandez et al., 2020). This highlights a general challenge for the field.

Given the plethora of known E3 ligases, it will be critical to define which of these are selectively involved in presynaptic autophagy, versus, for example, proteasomal and/or endolysosomal systems. Given the ability of these E3 enzymes to bind various E2s, this will be a daunting, but important challenge. Equally important will be the identification of their associated substrates. In the studies on synapses lacking Bassoon a poignant subset of SV proteins became hyper-ubiquitinated, but to what end? In another study, only a subset of SV proteins was selectively degraded by the endolysosomal system during high synaptic activity conditions (Sheehan et al., 2016), begging the question of which E2s and E3s direct these pathways of destruction.

While it is attractive to consider that there is a high specificity in the clearance systems for particular aspects of presynaptic proteostasis, it is far more likely that there is high flexibility and crosstalk between the different systems, including autophagy, endolysosomal or proteasomal degradation systems, that participate in the removal of different subsets of presynaptic proteins or organelles, like SVs, as was demonstrated for the degradation/turnover of other cellular systems. For example, ATG5-deficient neurons can survive and still display SV recycling (Negrete-Hurtado et al., 2020). Actually, they rather accumulate ER in axons and increase excitatory neurotransmission (Kuijpers et al., 2021) indicating flexibility and compensatory potential in the system.

From a functional perspective, there are several schools of thought: (i) that presynaptic autophagy primarily operates on a slow time scale for the basal removal of aging and/or defective proteins and membranous organelles, (ii) that it is directly involved in the destruction of SVs during high activity, and/or (iii) that it responds to eliminate proteins/membranes following acute and chronic stress or insults.

A key to solving some of these issues lies in how fast and with what specificity presynaptic autophagy can operate. Utilizing the light-activated free-radical generating protein SuperNova (SN) tethered to different SV proteins, it was recently shown that through the acute damage of these proteins, presynaptic autophagy could be induced in minutes and to depend on ubiquitination (Hoffmann et al., 2019). Here, the clearance mechanisms operated with high-specificity, directing the destruction of the SN-tagged protein (SN-synaptophysin or SN-synaptotagmin) to autophagy, but leaving other SV proteins in action. Intriguingly, such acute ROS-mediated damage had real-time effects on synaptic transmission only when autophagy was simultaneously inhibited with drugs or ATG5 knockdown (Hoffmann et al., 2019). One can thus conclude that the presynaptic autophagy machinery is capable of operating in real-time and can be highly selective, removing only damaged and ubiquitinated proteins ensuring the health and functionality of presynaptic boutons. Consistent with studies on boutons lacking Bassoon, these studies highlight the role of the ubiquitination system in tagging proteins for their elimination by autophagy. They also illustrate that the generation of ubiquitin-tagged damaged proteins is in and of itself a key driver of presynaptic autophagy. Potential cellular mechanisms associated with the sorting and delivery of these tagged proteins into newly formed phagophore membranes are discussed below. A point of consideration is the observation that, in boutons lacking Bassoon, autophagophore organelles contain seemingly intact SVs (Figure 5), indicating that whole SVs can also become cargos for autophagic destruction. From a ubiquitination perspective, this could be a consequence of the dramatic increase in the ubiquitination of many SV proteins due to the loss of Bassoon and the activation of Siah1 and Parkin, which then decorate the surface of SVs making them attractive as a cargo for engulfment (Hoffmann-Conaway et al., 2020).

## Cellular Mechanisms Governing Presynaptic Autophagy

The ability of presynaptic boutons to engage the autophagy degradative system in real-time to maintain its functionality, raise fundamental questions of how this is temporally and spatial achieved. At its most basic level, the ability of autophagy to operate in a spatially restricted manner requires the machinery to be present. Clearly there are two major arms to this process: (i) the sensing and tagging of misfolded or damaged proteins by ubiquitination, and (ii) the formation of autophagophore membranes capable of engulfing these cargos. As we have seen, some of the enzymes involved in the ligation of ubiquitin are scaffolded to components of the AZ cytoskeletal matrix, e.g., Bassoon and Piccolo (Waites et al., 2013; Okerlund et al., 2017; Hoffmann-Conaway et al., 2020).

Interestingly, many of the proteins critical for autophagophore formation are associated either with the perisynaptic endocytic zone, SVs or scaffold proteins of the AZ (Figure 4). Hints to how these might be linked together come from observations in *C. elegans* and *Drosophila* showing that there are hotspots of autophagosome formation in close proximity to or directly within presynaptic terminals (Soukup et al., 2016; Stavoe et al., 2016; Neisch et al., 2017; Vanhauwaert et al., 2017). Also, in mammals, many, but not all, constituents of the autophagic machinery have been detected reliably in synaptic boutons (Table 1). For example, of the Unc51-like kinases required for initiation of phagophore formation, only ULK3 has been detected in synaptosomes together with other components of the ULK complex, i.e., FIP100 or ATG101 (Taoufiq et al., 2020). This seems consistent with the observation that ULK1 and ULK2 are not essential for constitutive autophagy in the young murine brain (Wang et al., 2018) and hint to a function of ULK3 in (pre-)synaptic autophagy. Moreover, constituents of the PI3-Kinase C3 complex, including Vps34 and Beclin-1, have been found associated with SV preparations (Figure 3; Taoufiq et al., 2020). Further, there is compelling evidence that components of SV recycling interact with autophagy-related proteins (George et al., 2016; Soukup and Verstreken, 2017; Vanhauwaert et al., 2017), and core components of autophagosomes, including the membrane-delivering protein ATG9, are present in presynaptic boutons (Boyken et al., 2013; Stavoe et al., 2016). This suggests that the local biogenesis of autophagosomes could start at endocytic zones, following SV endocytosis or shortly thereafter when SV proteins pass through early and recycling endosome membranes, where quality control process sort out corrupted proteins.

Actually, membrane trafficking and protein sorting within boutons may be critical for autophagosome formation. Thus, the ATG9 is not only an integral component of the SV membrane (Boyken et al., 2013; Taoufiq et al., 2020), but is also the only transmembrane protein in the core machinery for autophagy (Lang et al., 2000; Noda et al., 2000; Young et al., 2006). A recent study deposited on the bioRxiv server actually suggests that SVs also directly may donate lipids to autophagosomes (Yang et al., 2020; Xuan et al., 2021). These authors propose that ATG9 is transported in vesicles generated at the trans-Golgi network to the presynaptic membrane, which undergo exo-endocytosis prior to accumulation at the peri-AZ and/or autophagosome formation. Mutations that prevent exocytosis (e.g., in *UNC13, UNC18* genes) or endocytosis (e.g., in *AP1, AP2*, or *SDPN-1/Syndapin 1* genes) negatively interfere with activity-dependent synaptic autophagy associated with the SV cycle (Imai et al., 2016; Yang et al., 2020; Xuan et al., 2021). Taken together the data suggest that membrane trafficking of ATG9 could couple the SV cycle to activity-dependent presynaptic autophagy. However, as mentioned above, SVs can also be recruited directly into autophagophores vi Rab26 and PLEKHG5 (Binotti et al., 2015; Lüningschrör et al., 2017; Figure 5).

How does neuronal activity and nutrient depletion affect presynaptic autophagy? Starvation/nutrient depletion usually induces autophagy via mTOR signaling (Bockaert and Marin, 2015). As stated above, it remains controversial whether and how strongly starvation affects presynaptic autophagy. At *Drosophila* larval NMJs, both starvation and increased neuronal activity induces ATG8 accumulation in presynaptic boutons (Soukup et al., 2016; Vanhauwaert et al., 2017). Similarly, mTOR inhibition by rapamycin induces autophagy at dopaminergic release sites and is associated with decreased SV numbers and evoked dopamine release (Hernandez et al., 2012). Furthermore, Catanese et al. (2018) report disorganized SV pools and rapid degradation of Bassoon upon nutrient limitation in rodent primary neuronal cultures. Re-supplementation of glucose can restore SV pools, but does not rescue Bassoon levels. In contrast, there are multiple studies reporting no or only minor effects on presynaptic autophagy (Mizushima et al., 2004; Maday and Holzbaur, 2016). The relative resistance of some neurons to autophagy induction in the presynaptic/axonal compartment by starvation/nutrient depletion may have various reasons that should be tested in future: one may be that presynaptic mTOR signaling is dominated by mTORC2 (McCabe et al., 2020), which is not linked to starvation-induced autophagy; another one could be that presynaptic autophagy (partly) depends on ULK3 (see above), while amino acid starvation-induced autophagy is controlled by ULK1/2 (Cheong et al., 2011) in a subset of neurons.

There is strong evidence for neuronal activity being a major factor regulating presynaptic autophagy. Thus, it is known that neuronal activity, and the recycling of SVs carrying key autophagic molecules, might contribute to the assembly of autophagosomes within boutons. Consistently, KCl stimulation has been shown to induce axonal autophagy and enhances retrograde autophagy flux in hippocampal primary neurons (Wang et al., 2015). Of note, activity can also engage the endolysosomal/ESCRT system to degrade subsets of SV protein (Sheehan et al., 2016).

At the organismal level, various studies imply that autophagy has a major impact on learning and memory (Shehata and Inokuchi, 2014; Liang and Sigrist, 2018; Hwang et al., 2019; Liang, 2019). However, in most cases it is impossible to assign these effects to neurons or even neuronal compartments where the relevant autophagic effect on memory formation is expressed. A particular case of presynaptic autophagic dysfunction seems to be the *tambaleante (tbl)* mouse, where the HERC1 E3 ubiquitin ligase is mutated. Among other phenotypes these mice display poor performance hippocampus-dependent learning including novel-object recognition, T-maze and Morris water maze tests (Montes-Fernandez et al., 2020; Perez-Villegas et al., 2020). In fruit flies, a tight association between age-dependent memory impairment and structural changes in presynaptic AZs has been observed. Both structural changes and memory decline could be counteracted by treatment with the autophagy enhancer spermidine (Gupta et al., 2016; Liang and Sigrist, 2018). Finally, conditional knockout of the presynaptic AZ protein Bassoon only in excitatory neurons of the murine forebrain, which is supposed to cause enhanced autophagy exclusively in glutamatergic terminals, causes improved memory performance in dentate gyrus-dependent learning tasks, such as contextual fear memory or spatial pattern separation (Annamneedi et al., 2018). This memory improvement was associated with the maintenance of juvenile synaptic plasticity at relevant performant path to dentate gyrus synapses. These examples support the view that well-functioning presynaptic autophagy is a prerequisite for maintaining brain synapses plastic and healthy.

## Potential Scenario for the Regulation of Presynaptic Autophagy

An important unresolved question is how basal, activity-dependent and protein damage induced autophagy relate to each other? One potential answer is they are unrelated. A more parsimonious answer is they are intimately linked playing complementary and sometimes redundant roles. Moreover, the transition between them may primarily relate to the rate of flux and the pathways activated. Given the available data and emerging concepts in the field, we can conceive the following scenario for ubiquitination-dependent modes of protein turnover. It begins with a few knowns. First, that productive autophagy requires the generation of poly-ubiquitin tagged cargos (Figure 5), which can occur during use and natural aging of SVs and their associated proteins or through their acute damage, e.g., by ROS or nitrosylation among others. This could also occur during development, e.g., during synaptic pruning and/or trophic factor withdrawal via the E3 ligase MYCBP2/RPM-1/Highwire (Grill et al., 2016; Crawley et al., 2019). In principle, all this would be mediated by ubiquitin ligases, only a few of which are currently known. Second, that the degradation of these cargos requires the generation of phagophore membranes, which through adaptor proteins such as p62/SQSTM1, recruit the ubiquitinated cargos (Figure 5). This in turn requires the presence and activation of the machinery required to create the phagophore membrane. While there is a clear interdependency of these two arms of autophagy, as disruption of either blocks presynaptic autophagy, it is unclear how this is coordinated, regulated and/or what sensors are used. An attractive focal point for the generation of phagophore membranes and the sorting of ubiquitin-tagged cargos within presynaptic boutons are the early and sorting/recycling endosomal compartments, which maybe be used during basal, activity-dependent and protein damage induced autophagy. Here, it is well appreciated that these membranes operate as quality control stations, working to regenerate functional SVs with the correct complement of proteins. As such, it is easy to imagine that ubiquitin tagged proteins destined for destruction would be sorted into autophagic cargo vesicles at this point. Similarly, it would be a natural site for sorting the autophagy machinery (ATG9 among others), normally associated with SV-like vesicles, into precursor phagophore membranes that then mature into autophagosomes decorated with ATG8-like molecules (LC3, GABARAPs). In the event that there is little cargo, ATG9 would mostly be recycled back into the existing pool of SV-like membranes. Given the complexity of membrane trafficking, it would be important to entertain that many membranes could be used to generate autophagosomes and/or carry ubiquitinated cargos, including the plasma membrane, other endosomal compartments and/or the ER and potentially also SVs.

## Further Open Questions

It is well documented that accurate performance of all types of autophagy as well as the endolysosomal system are of utmost importance for cellular health. This applies particularly to neurons and their synapses with their extremely long lifespans. All major brain disorders are associated with failures of these clearance systems, either as causes or consequences of the pathology. In particular, neurodegenerative disorders, such as Parkinson’s Disease or Alzheimer’s can be directly linked with autophagy failures, though physiological aging is also associated with slow exhaustion of clearing systems. We have largely ignored this aspect in the present article and focused mainly on the molecular inventory of the presynapse, because disease-related issues have been the subject of numerous review articles during recent years (e.g., Rubinsztein, 2006; Mizushima et al., 2008; Bezprozvanny and Hiesinger, 2013; Michel et al., 2016; Deng et al., 2017; Menzies et al., 2017; Dikic and Elazar, 2018; Liang and Sigrist, 2018; Soukup et al., 2018; Lee and Kim, 2019; Malik et al., 2019; Pan et al., 2019; Giovedi et al., 2020; Tomoda et al., 2020). However, it is very obvious that there are multiple gaps in our knowledge about disease mechanisms. Therefore, a detailed understanding of the molecular organization of (pre-)synaptic protein turnover and clearance systems at the nanoscale is a major future challenge for the field.

Additional questions worth consideration include: Do clearance systems operate in a similar manner at all synapses? Do autophagic events occur near/within boutons or more remotely? On what time scales does autophagy operate? Which ubiquitin tagging systems (E2, E3 enzymes) are utilized? How many and when are the different lipidated ATG8-like conjugates used? Are different Unc-51-like kinase employed under specific conditions? What autophagy receptors/cargo adaptors are tapped and at which synapses? What ubiquitin-independent mechanisms are in operation?

Further questions concern the sorting and routing of damaged proteins. Where does the sorting take place? Which organelles contribute membranes to the phagophore? Do SVs contribute to phagophore formation? Can SVs even act as a starting point for phagophore assembly? How can clearance pathways compensate for each other and is this controlled and regulated?

Finally, there are interesting recent observations that liquid phase separation might also play a role in PAS formation and the initiation of autophagy (Fujioka et al., 2020; Hollenstein and Kraft, 2020). For example, evidence from yeast indicates that the endocytic protein Ede1 (the yeast homolog of p115/Uso1) can mediate the formation endocytic protein condensates (ENDs) that then enter an autophagic degradation path (Wilfling et al., 2020). Another recent study revealed that condensed liquid droplets, formed from the AZ proteins RIM, RIM-binding protein and ELKS/CAST2 can tether SVs *in vitro* (Wu et al., 2021). The authors hypothesize that similar processes play a role in organelle biogenesis and autophagy. It will thus be interesting to see whether phase separation indeed triggers autophagy at the perisynaptic endocytic zone.

## Author Contributions

EG, CG, and MK wrote the article. AK designed and prepared the figures. RP provided bioinformatic and database analyses including Figure 2. All authors contributed to the conceptualization and the discussion of the content.

## Conflict of Interest

The authors declare that the research was conducted in the absence of any commercial or financial relationships that could be construed as a potential conflict of interest.

## Publisher’s Note

All claims expressed in this article are solely those of the authors and do not necessarily represent those of their affiliated organizations, or those of the publisher, the editors and the reviewers. Any product that may be evaluated in this article, or claim that may be made by its manufacturer, is not guaranteed or endorsed by the publisher.

## Footnotes

1
www.synprot.de

2
www.syngoportal.org

## Abbreviations

AZ: active zone
BAR domain: Bin-Amphiphysin-Rvs domain
CMA: chaperone-mediated autophagy
NMJ: neuromuscular junction
PAS: pre-autophagosomal structure (also: phagophore assembly site)
Peri-AZ: periactive zone, = main endocytic zone
SV: synaptic vesicle
SN: SuperNova
S/T: Serine/Threonine
UPS: Ubiquitin-Proteasome-System.
